# Biomechanics of In-Stance Balancing Responses Following Outward-Directed Perturbation to the Pelvis During Very Slow Treadmill Walking Show Complex and Well-Orchestrated Reaction of Central Nervous System

**DOI:** 10.3389/fbioe.2020.00884

**Published:** 2020-07-28

**Authors:** Zlatko Matjačić, Matjaž Zadravec, Andrej Olenšek

**Affiliations:** Research and Development Unit, University Rehabilitation Institute Republic of Slovenia, Ljubljana, Slovenia

**Keywords:** perturbed walking, medio-lateral ankle strategy, inertial strategy, braking strategy, stepping strategy

## Abstract

Multiple strategies may be used when counteracting loss of balance during walking. Placing the foot onto a new location is not efficient when walking speed is very low. Instead medio-lateral displacement of center-of-pressure, rotation of body segments to produce a lateral ground-reaction-force, and pronounced braking of movement in the plane of progression is used. It is, however, presently not known in what way these in-stance balancing strategies are interrelated. Twelve healthy subjects walked very slowly on an instrumented treadmill and received outward-directed pushes to the waist. We created experimental conditions where the use of stepping strategy to recover balance following an outward push was minimized by appropriately selecting the amplitude and timing of perturbation. Our experimental results showed that in the first part of the response the principal strategy used to counteract the effect of a perturbing push was a short but substantial increase in lateral ground-reaction-force. Concomitant slowing of the movement and related anterior displacement of center-of-pressure enabled lateral displacement of center-of-pressure which was, together with a short but substantial increase in vertical ground-reaction-force, instrumental in reducing the inevitable increase of whole-body angular momentum in the frontal plane. However, anterior displacement of center-of-pressure and increased vertical ground-reaction-force also induced an increase in whole-body angular momentum in the sagittal plane. In the second part of the response the lateral ground-reaction-force was decreased with respect to unperturbed walking thus allowing for a decrease of whole-body angular momentum in the frontal plane. Additionally, an increase in anterior ground-reaction-force in the second part of the response propelled the center-of-mass in the direction of movement, thus re-synchronizing it with the frontal plane component of the center-of-mass as well as decreasing whole-body angular momentum in the sagittal plane. The results of this study show that use of in-stance balancing strategies counteracts the effect a perturbing push imposed on the center-of-mass, re-synchronizes the movement of center-of-mass in sagittal and frontal planes to the values seen in unperturbed walking and maintains control of whole-body angular momentum in both frontal and sagittal planes.

## Introduction

Appropriate reactions to unexpected perturbations, particularly those acting in the medio-lateral plane, are essential for stable walking. Several studies have applied outward- and inward-directed pushes to the pelvis of walking subjects to investigate the repertoire of dynamic balancing responses (Hof et al., 2010; Matjačić et al., 2017; Vlutters et al., 2018b). While responses to inward perturbations are fairly uniform across walking speeds and perturbation parameters and show differences mainly in relation to the instance of the gait cycle at which the perturbation occurs, the responses to outward perturbations are more complex (Matjačić et al., 2019). These depend on several factors including the magnitude of the perturbation, the instance of the gait cycle at which the perturbation occurs and walking speed. In this study, we were focused solely to outward perturbations. The majority of studies have investigated balancing responses following outward perturbations at walking speeds normally used by able-bodied subjects (around 1.2 m/s). Foot placement adjustment of the swinging leg, termed “stepping strategy,” was identified as the most important strategy to recover balance following perturbation (Rankin et al., 2014; Wang and Srinivasan, 2014; Afschrift et al., 2018; Bruijn and van Dieen, 2018). Studies have also shown that corrective action following a perturbation starts earlier in the form of displacement of COP under the stance leg in the direction of the perturbation, termed “medio-lateral ankle strategy” (Hof and Duysens, 2018; Reimann et al., 2018). In accordance with the inverted pendulum model lateral COP displacement increases the horizontal component of GRF thus opposing the action of the perturbation (Hof, 2007). The third strategy called “inertial strategy” is related to rotation of limb segments (Bruijn and van Dieen, 2018; van den Bogaart et al., 2020). This can be for example rotation of the trunk (Horak and Nashner, 1986) or arm and leg movements that are observed when walking on a narrow beam (Chiovetto et al., 2018). Studies that investigated balancing responses following moderate lateral perturbing pushes at the waist with a range of walking speeds (0.8–1.2 m/s) have not identified noticeable arm, leg or trunk motions. The fourth strategy used after the perturbation in the frontal plane is related to the slowing-down of movement in the plane of progression which has been termed “braking strategy” (Matjačić et al., 2017; Hof and Duysens, 2018; Vlutters et al., 2018b).

Several studies looked into how humans react to perturbing pushes at slower walking speeds where stepping strategy may be inefficient due to the relatively long period of time required for the next step to occur (Olenšek et al., 2016; Matjačić et al., 2017, 2019; Vlutters et al., 2018b). Taking corrective action by adequately placing a swinging leg onto a new location takes time and is dependent on the instant during the gait cycle when the perturbation commenced (Hof et al., 2010). For example, if a perturbation occurs in the beginning of the stance phase the whole step time will lapse before a corrective action originating from the “stepping strategy” can begin. Also, use of the “stepping strategy” is progressively diminished while the use of hip abductors of the stance leg, that increase the GRF in the direction opposite to the action of the perturbation, is progressively increased as walking speed is reduced (Matjačić et al., 2018). In our recent perturbation studies (Matjačić et al., 2018, 2019), one of the walking speeds examined was 0.4 m/s, which is frequently the speed practiced by people following a stroke (Raja et al., 2012), we have observed a pronounced impulse-like response in the medio-lateral component of GRF under the stance leg following an outward push that was sufficient to stabilize the body in the lateral plane so that the location of the following step did not differ from the one during unperturbed walking, thus rendering use of the “stepping strategy” unnecessary in the subsequent step.

As walking speed decreases from 0.8 to 0.4 m/s the “braking strategy,” characterized by increasingly larger anterior COP displacement and increased braking action of GRF in the sagittal plane following an outward perturbation, is used increasingly (Matjačić et al., 2017, 2019). The origin and functional role of this “braking strategy” are currently unknown. Hof and Duysens (2018) and Vlutters et al. (2018b) have observed a pronounced co-contraction of ankle joint muscles while Vlutters et al. (2018b) also observed co-contraction of the knee and hip joint muscles in the stance leg immediately after an outward push. They have suggested that this co-contraction, which is more pronounced at lower walking speeds, increases the mechanical impedance of joints, thus (i) temporarily slowing movement in the plane of progression and (ii) increasing the stability of the stance leg in the vertical direction. Hof and Duysens (2018) have further suggested that reducing the forward velocity allows the brain to evaluate external conditions before taking action. It remains unclear why use of the “braking strategy” following an outward perturbation increases with decreased walking speed (Matjačić et al., 2019).

Several studies have highlighted that whole-body angular momentum (H), calculated around the COM, is a quantity that is tightly regulated during human walking. During steady-state walking changes of angular momentum undergo relatively small oscillations which are balanced across the gait cycle (Herr and Popovic, 2008). This implies that the dynamics of COP and GRF are such that fluctuations of a derivative of H (dH/dt) are minimized throughout the gait cycle. Previous studies by Hof et al. (2010), Vlutters et al. (2018b), and Matjačić et al. (2017) have shown that dynamic balancing responses following outward-directed waist perturbations during walking at normal speeds as well as at lower speeds do not incorporate noticeable head, arm, and trunk movement, suggesting that tight control of angular momentum in sagittal and frontal planes is also exercised during perturbed walking. Walking on a narrow beam that requires a considerable share of “inertial strategy” reflected in movements of the trunk, arms, and legs has also shown remarkably small variations in whole body angular momentum (Chiovetto et al., 2018). It, however, remains unclear whether tight control of whole-body angular momentum is also of high priority during very slow walking. Bruijn and van Dieen (2018) have in their recent review pointed out that it is to a large extent unclear how the balancing strategies that primarily change the COP and GRF under the leg in stance interact and what the governing principles of their interaction are.

**FIGURE 1:**
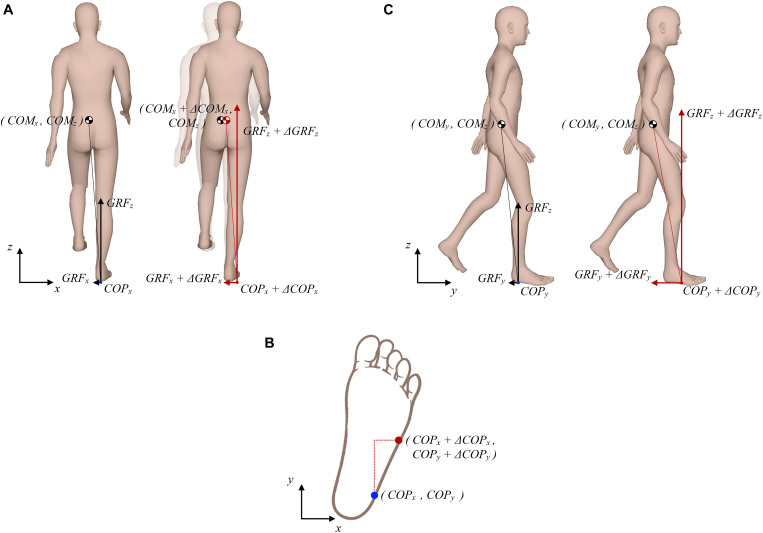
Theoretical considerations on possible interplay between COP, GRF, and COM that produces zero change in the whole-body angular momentum. **(A)** Frontal plane situation; left side shows the unperturbed situation, right side shows the situation following a perturbing push to the right. **(B)** Schematic presentation of how the lateral COP can be further displaced if the anterior COP is increased. **(C)** Sagittal plane situation; left side shows the unperturbed situation, right side shows the situation following a perturbing push to the right.

Figure 1 outlines purely theoretical considerations on how COM, COP, and GRF determine changes in angular momentum around COM during the single stance of walking. Figure 1A (left side) depicts a purely hypothetical situation in the frontal plane where instantaneous magnitudes of GRFz and GRFx are such that at given COPx displacement, viewed relative to COMx, GRF passes exactly through COMx thus producing zero change in angular momentum (dHy/dt). If COMx is displaced to the right as a consequence of an outward perturbation (Figure 1A, right side) an increase in GRFx is required at very slow walking pace to act against the perturbation as shown by Matjačić et al. (2019). Such an increase in GRFx would result in a change of angular momentum in the frontal plane if not appropriately balanced (van den Bogaart et al., 2020). Therefore, concomitant changes in COPx and GRFz would be needed to oppose the effect of increased GRFx. COPx would need to be displaced further to the right to increase the moment arm of GRFz. However, given the foot geometry, possibilities to displace COPx further to the lateral edge of the foot are rather limited. Thus, adequate increase in GRFz would also be required to oppose the effect of increased GRFx on the change of angular momentum. Furthermore, taking into consideration foot geometry and typical foot orientation during stance, which is rotated outward by approximately 10 degrees (Bonnefoy-Mazure and Armand, 2015), COPx can be further displaced toward the edge of the foot if COPy is displaced forward as indicated in Figure 1B, which have implications for control of angular momentum in the sagittal plane. Figure 1C (left side) shows a purely hypothetical situation in the sagittal plane where instantaneous magnitudes of GRFz and GRFy are such that at a given COPy displacement, viewed relative to COMy, the resultant GRF passes exactly through COMy. However, considering the assumed changes made to COPy and GRFz, as suggested in Figures 1A,B, an appropriate change also needs to be made in GRFy in order to maintain zero change of angular momentum (dHx/dt) as shown in Figure 1C (right side). This increase in GRFy will, however, as a consequence have a “braking” effect on COMy movement.

This purely theoretical considerations do not entirely apply to human walking where GRF, COP, and COM are never in perfect equilibrium. In fact, when “inertial strategy” is used to restore balance this may result in a change of angular momentum (van den Bogaart et al., 2020). Furthermore, our theoretical consideration would require almost doubling the GRFz in order to maintain zero change angular momentum, which is not very likely as this would mean excessive acceleration of COMz which would ultimately stop with full knee extension making such an excessive increase of GRFz entirely inefficient. Our theoretical consideration further suggests substantial increase in the braking action of GRFy, which is also not very likely as this would substantially reduce forward progression. It appears that maintaining tight regulation of angular momentum following an outward perturbation during very slow walking may be a particularly demanding task and may involve more complex mechanisms than the one considered in Figure 1. Nevertheless, the simple model presented in Figure 1 indicates the direction in which we can expect changes in GRF, COP, and COM which would be required to minimize the changes of angular momentum being induced by the experimentally observed GRFx force impulse (Matjačić et al., 2019) that is generated as a response to an outward perturbation.

The aim of this study was to investigate organization of dynamic balancing responses following outward perturbation applied during the double stance at very low walking speed within the framework of conservation of overall angular momentum around COM. Our first hypothesis was that an impulse-like increase of GRFx that rapidly acts against an outward perturbation is accompanied by concomitant changes in GRFz, GRFy, COPx, and COPy that according to the model presented in Figure 1 act to maintain tight regulation of the overall angular momentum around the COM in the sagittal and frontal planes. We further hypothesized that movement in the direction of progression would be temporarily slowed due to the anticipated increase in GRFy.

## Materials and Methods

### Subjects

Twelve healthy males without known history of neuromuscular or orthopedic problems (age: 32.9 ± 6.9 years, height: 179.2 ± 3.7 cm, and mass: 78.8 ± 6.6 kg) participated in this study after signing informed-consent forms in accordance with the Declaration of Helsinki. The study was approved by the Slovenian National Ethics Committee.

### Instrumentation

**FIGURE 2:**
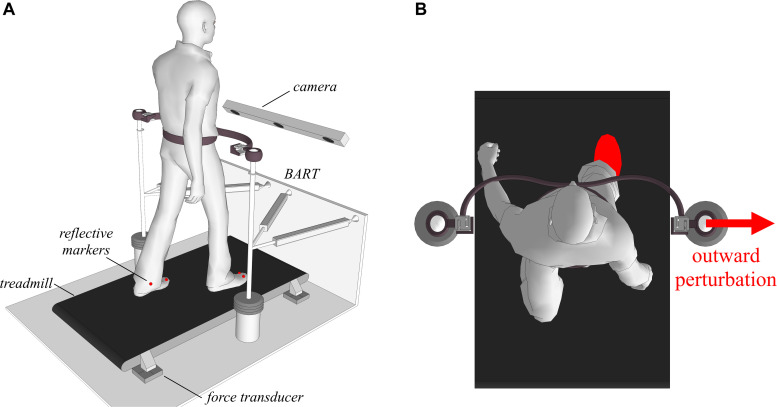
Subjects walked on an instrumented treadmill, while receiving pelvic perturbations through the BART device in inward and outward directions when entering the stance with either the left or the right leg. **(A)** A graphical illustration of the experimental setup. **(B)** A graphical illustration of an outward perturbation occurring at right heel strike.

Figure 2 shows a schematic of the experimental environment, which consisted of a balance-assessment robot (BART) and an instrumented treadmill. Here only a brief description of the experimental setup is given, as a more detailed description is provided elsewhere (Olenšek et al., 2016; Matjačić et al., 2017). The BART interfaces with the pelvis of a walking participant with 6 degrees of freedom (DOF). Five of the DOFs (translation of the pelvis in the sagittal, lateral, and vertical directions; pelvic rotation and pelvic list) are actuated and admittance-controlled (control loop running at 1 kHz), so providing transparent haptic interaction with negligible power transfer (Matjačić et al., 2017). The sixth DOF (pelvic tilt) is passive. The BART is capable of delivering perturbations in the forward/backward and inward/outward directions. In this study, we only considered outward perturbations delivered in the frontal plane with the right leg entering the stance as depicted in Figure 2.

Center-of-mass movement was estimated according to the sacral marker method (Gard et al., 2004; Yang and Pai, 2014; Jeong et al., 2018) from the translational movement of the subjects’ pelvis, which was assessed from the movement of the BART, similarly as in our previous studies (Matjačić et al., 2017, 2018). Recordings of GRF and COP were obtained by means of four precision force transducers (K3D120, ME Systeme GmbH, Hennigsdorf, Germany) placed underneath the treadmill. Foot kinematics data were assessed by means of an Optitrack camera (Trio V120, NaturalPoint Inc., Corvallis, OR, United States). Kinematic data from one subject were excluded due to equipment malfunction. Passive reflective markers were placed on the participants’ feet (on the medial malleoli, and the first and fourth metatarsal joints). Sampling frequency for the kinematic and kinetic data was 50 Hz which is considered to be adequate for this type of study (Rhea et al., 2015).

### Experimental Protocol

The experimental protocol is substantially similar to the one used in our recent study (Matjačić et al., 2019). First, subjects walked at a treadmill speed set to 0.4 m/s for a period of three minutes. This was followed by a period of around half an hour of perturbed walking. Perturbations were delivered with a randomly varied pause that ranged from 6 to 8 s in order to avoid predictability of the perturbation occurrence. Two perturbation directions, outward and inward relative to the leg in stance, three perturbation onsets (at 0, 30, and 60% of the stance phase of a gait cycle) and three perturbation amplitudes (5, 10, and 15% of body weight) were varied. Each combination of perturbation parameters was repeated seven times. This yielded a total of 252 perturbing pushes that were block-randomized. Perturbations took the form of a force impulse lasting 150 ms similar to our previous studies (Olenšek et al., 2016; Matjačić et al., 2017, 2018). Prior to this study all subjects visited our laboratory where they practiced unperturbed and perturbed walking on the BART system for approximately half an hour.

### Measurements and Data Analysis

Although we assessed postural responses at three levels of perturbation onset and at three perturbation strengths, in further analysis we included only outward perturbations that commenced at 0% of the gait cycle and with a perturbation strength of 10% of body weight.

The COM, COP, GRF, and ankle kinematics data were first segmented into strides with the gait cycle defined as the period between two consecutive right heel strikes, as detected from COPx and COPy signals. One gait cycle after the onset of perturbation, was analyzed representing the perturbed experimental condition. Similarly, one gait cycle was analyzed where no perturbation occurred representing the unperturbed experimental condition. The data of the gait cycle were split to the in-stance period (from right heel strike to the next left heel strike) and to the stepping period (from left heel strike to the next right heel strike). The data in both periods was normalized to the duration of each period for unperturbed and perturbed experimental conditions separately thus allowing for direct comparison between different subphases of the in-stance and stepping periods of the gait cycle. The estimate of COMx for the perturbed experimental condition was adjusted using the weighted mass method (Winter, 2009). One mass was represented by the left leg that was in swing phase following the perturbation and displayed marked abduction in comparison to the unperturbed experimental condition, while the second mass was represented by the remaining of the body. The center-of-mass position of the left leg was determined from measurements of pelvic position and left ankle marker position where centre-of-mass and mass for upper and lower leg values were estimated from anthropometric tables (Winter, 2009). Changes of whole-body angular momentum (dHx/dt and dHy/dt) were calculated with the following two equations (Neptune and McGowan, 2011; Neptune and McGowan, 2016):

(1) dHy/dt=-GRFx*COMz-GRFz*(COPx-COMx)

(2) dHx/dt=GRFz*(COPy-COMy)+GRFy*COMz

COM, COP, GRF, ankle kinematics, dHx/dt, and dHy/dt signals were averaged across seven repetitions for each subject for both experimental conditions. If any of the seven repetitions markedly differed it was excluded from averaging. COM, COP, GRF, and dH/dt were subsequently made dimensionless following the procedure proposed by Hof (1996). Duration of in-stance and stepping periods was normalized by the duration of unperturbed gait cycle in each individual subject. COM, COP, and ankle marker data were normalized by the height of each individual subject, GRF data were normalized by the weight of each individual subject while dH/dt data were normalized by the weight and height of each individual subject. Group means and standard deviations were built over the twelve subjects for unperturbed and perturbed experimental conditions using the calculated means of each individual subject. Group means and standard deviations were calculated for durations of in-stance and stepping periods of the gait cycle for both experimental conditions. Group means and standard deviations were calculated for dHx/dt and dHy/dt summed over the in-stance and stepping periods for both experimental conditions.

### Statistical Analysis

Normal distribution of data was tested using a Kolmogorov–Smirnov test. To assess the differences in COM, COP, GRF, ankle kinematics, dHx/dt and dHy/dt signals Statistical Parametric Mapping – SPM for one-dimensional signals (Pataky, 2010) was used to test for the differences between unperturbed and perturbed experimental conditions. Paired *t*-test was used to test for the differences between the durations of in-stance and stepping periods as well as for summed dHx/dt and dHy/dt between unperturbed and perturbed experimental conditions. *P* values less than 0.05 were considered to be significant. All calculations were performed in MATLAB R2018b (The MathWorks, Inc., Natick, MA, United States).

## Results

Dynamic balancing responses to outward perturbations were substantially similar for both sides. In continuation we provide results where the right leg was the stance leg during the perturbations as shown in Figure 2.

### Duration of In-Stance and Stepping Periods of Responses

**FIGURE 3:**
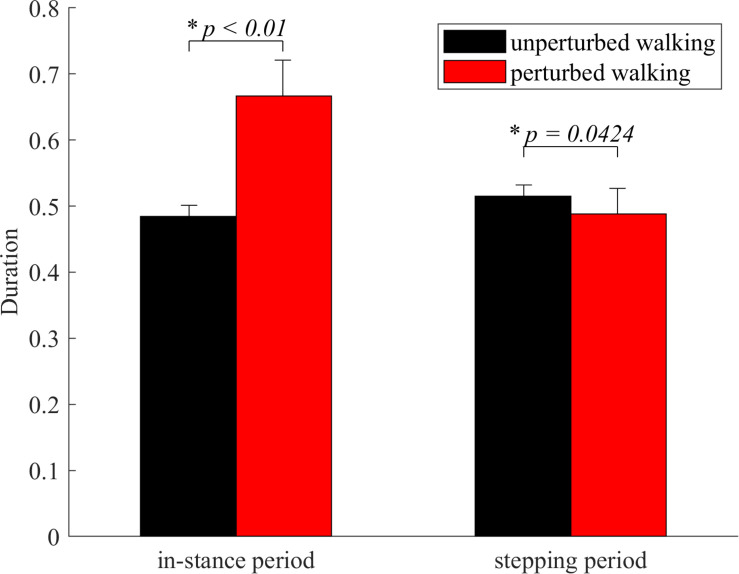
Dimensionless group average and standard deviations of in-stance and stepping periods durations for both experimental conditions. Statistical significance is indicated with an asterisk (^∗^).

In-stance period was significantly longer in perturbed experimental condition compared to the unperturbed experimental condition as shown in Figure 3. Stepping period was significantly shorter in perturbed experimental condition compared to the unperturbed experimental condition as shown in Figure 3.

### Center-Of-Mass and Ground-Reaction-Forces

**FIGURE 4:**
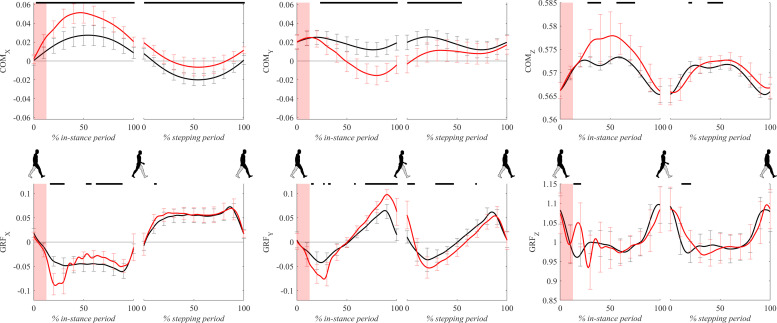
Dimensionless group-average COM and GRF responses after outward perturbations (*N* = 12, except for COMx where *N* = 11). Black corresponds with the unperturbed experimental condition, red with perturbed experimental conditions. Standard deviations are shown at each 10% of a gait cycle. One gait cycle divided into the in-stance and stepping periods, normalized to its duration separately for each experimental condition, is shown following a perturbing push. To visualize normalization of both periods short discontinuity is introduced on abscise. The perturbation period is marked with the red bar. Periods where statistically significant changes were determined between both experimental conditions are indicated with piece-wise continuous black lines on top of each graph.

Figure 4 shows COM and GRF signals for both experimental conditions. Following the perturbation COMx displayed significant lateral displacement with respect to unperturbed walking throughout the whole gait cycle. GRFx during perturbed walking showed amplitudes around 20% of the in-stance period which were significantly higher than unperturbed values in the direction opposite to COMx movement. From 40 to 100% of the in-stance period GRFx significantly dropped with respect to unperturbed values.

Following the perturbation COMy showed significant slowing of the movement with respect to unperturbed walking throughout the majority of the gait cycle. GRFy during perturbed walking showed amplitudes around 20% of the in-stance period which were significantly higher than unperturbed values in the direction opposite to walking progression. Significant differences were also displayed between approximately 80–100% of the in-stance period where GRFy in perturbed walking showed significantly higher amplitudes with respect to the unperturbed values in the direction of walking. There were also significant differences between the experimental conditions in the middle of the stepping period where GRFy in perturbed walking show higher amplitudes in the direction opposite to walking progression.

Following the perturbation COMz showed slight but significant upward displacement with respect to the unperturbed values in the middle of the in-stance period. Immediately after the perturbation finished GRFz showed a significant increase with respect to the unperturbed values for a short period around 20% of the in-stance period. GRFz assessed in perturbed walking was significantly higher also for a short period in beginning of the stepping period.

### Center-Of-Pressure

**FIGURE 5:**
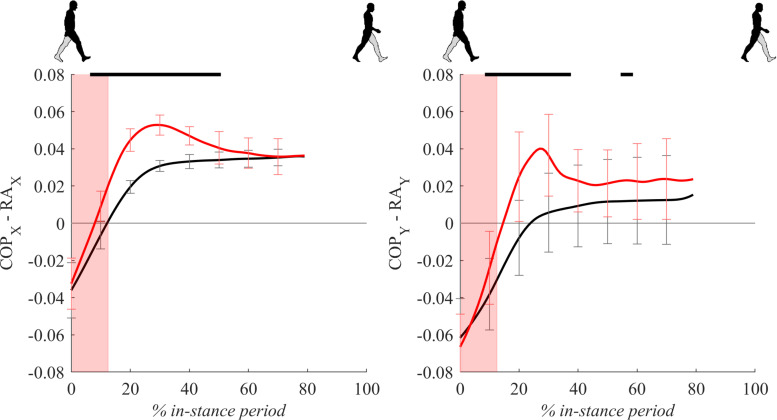
Dimensionless group-average COP responses relative to the right ankle (medial malleolus) marker position (COPx – RAx and COPy – RAy) after outward perturbations (*N* = 11). Standard deviations are shown at each 10% of a gait cycle. Black corresponds with the unperturbed experimental condition, red with perturbed experimental conditions. In-stance period, normalized to its duration separately for each experimental condition, is shown following a perturbing push. The perturbation period is marked with the red bar. Periods where statistically significant changes were determined between both experimental conditions are indicated with piece-wise continuous black lines on top of each graph.

Figure 5 shows COP displacements relative to the ankle position for the in-stance period. Following the perturbation, significant displacements of COPx and COPy relative to the position of the ankle marker can be seen in the period from 10 to 50% of the in-stance period with peaks approximately coinciding with the observed peaks in GRFx and GRFy.

**FIGURE 6:**
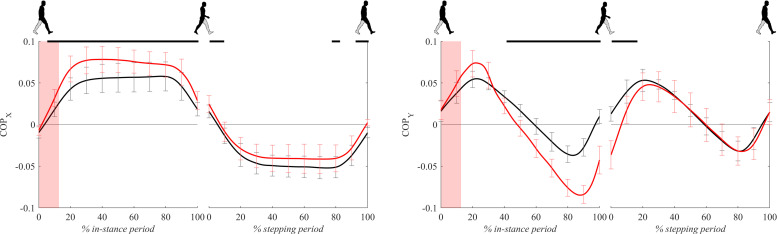
Dimensionless group-average COP responses after outward perturbations (*N* = 12). Standard deviations are shown at each 10% of a gait cycle. Black corresponds with the unperturbed experimental condition, red with perturbed experimental conditions. One gait cycle divided into the in-stance and stepping periods, normalized to its duration separately for each experimental condition, is shown following a perturbing push. To visualize normalization of both periods short discontinuity is introduced on abscise. The perturbation period is marked with the red bar. Periods where statistically significant changes were determined between both experimental conditions are indicated with piece-wise continuous black lines on top of each graph.

Figure 6 shows COP signals for both experimental conditions. Following the perturbation COPx displayed significantly larger lateral displacement with respect to unperturbed walking throughout the majority of in-stance and stepping periods.

Following the perturbation COPy displayed significant anterior displacement with respect to unperturbed walking around 20% of the in-stance period. Later, from 40 to 100% of the in-stance period and in the first 20% of the stepping period COPy in perturbed walking showed significantly posterior displacement with respect to unperturbed walking.

**FIGURE 7:**
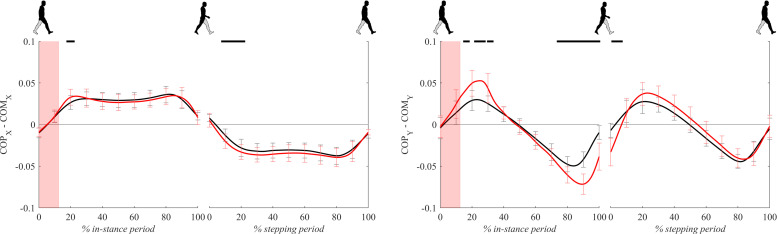
Dimensionless group-average COP-COM responses after outward perturbations (*N* = 11 for *x* direction and *N* = 12 for *y* direction). Standard deviations are shown at each 10% of a gait cycle. Black corresponds with the unperturbed experimental condition, red with perturbed experimental conditions. One gait cycle divided into the in-stance and stepping periods, normalized to its duration separately for each experimental condition, is shown following a perturbing push. To visualize normalization of both periods short discontinuity is introduced on abscise. The perturbation period is marked with the red bar. Periods where statistically significant changes were determined between both experimental conditions are indicated with piece-wise continuous black lines on top of each graph.

Figure 7 shows COP – COM signals (the distance between the COP and the COM projected to the ground) for both experimental conditions. COPx – COMx signals, which are essentially medio-lateral moment arms for the GRFz in Eq. 1, were mostly comparable between both experimental conditions except for short initial periods of in-stance and stepping periods where COPx – COMx for perturbed walking was significantly higher with respect to unperturbed walking.

COPy – COMy signals, which are essentially antero-posterior moment arms for the GRFz in Eq. 2, showed substantially larger values for perturbed experimental condition from 20 to 40% and 80 to 100% of the in-stance period as well as in the first half of the stepping period.

### Changes of Whole-Body Angular Momentum

**FIGURE 8:**
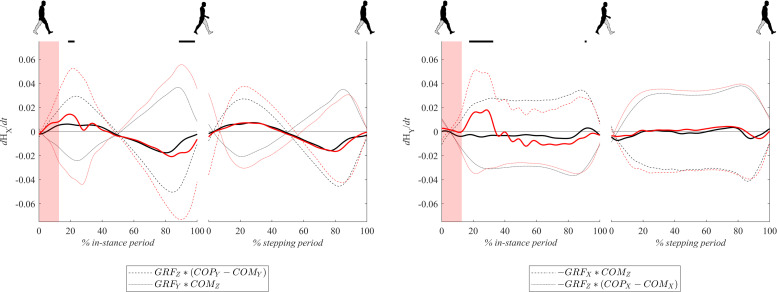
Dimensionless group-average dH/dt (solid lines) responses after outward perturbations (*N* = 12 for *x* direction and *N* = 11 for *y* direction). Dashed and dotted lines show individual components contributing to dH/dt as indicated in the legend. Black corresponds with the unperturbed experimental condition, red with perturbed experimental conditions. One gait cycle divided into the in-stance and stepping periods, normalized to its duration separately for each experimental condition, is shown following a perturbing push. To visualize normalization of both periods short discontinuity is introduced on abscise. The perturbation period is marked with the red bar. Periods where statistically significant changes were determined between both experimental conditions are indicated with piece-wise continuous black lines on top of each graph.

Figure 8 shows dH/dt signals for both experimental conditions. Changes of whole body-angular momentum in the frontal plane dHy/dt following perturbations were significantly larger in the period from 20 to 40% of the in-stance period in comparison to the unperturbed experimental condition. This was predominantly due to the significantly increased GRFx^∗^COMz component while the GRFz^∗^(COPx – COMx) component remained similar to the values observed in the unperturbed experimental condition. In the remaining part of the in-stance period dHy/dt was smaller in the perturbed experimental condition with respect to values in the unperturbed experimental condition. This was predominantly due to the substantially decreased GRFx^∗^COMz component while the GRFz^∗^(COPx – COMx) contribution remained similar to values observed in the unperturbed experimental condition.

Changes of whole-body angular momentum in the sagittal plane dHx/dt following perturbation were significantly larger in the period around 20% of the in-stance period in comparison to the unperturbed experimental condition while in the period from 90 to 100% of the in-stance period they were significantly smaller. Individual components of Eq. 2, GRFz^∗^(COPy – COMy) and GRFy^∗^(COMz) were both significantly larger following perturbation, which was predominantly due to the increase in COPy and GRFy. However, the influence of the former was greater both in the first part of the stance phase as well as in the second part. Since both contributions changed signs at midstance their sum effectively canceled out.

**FIGURE 9:**
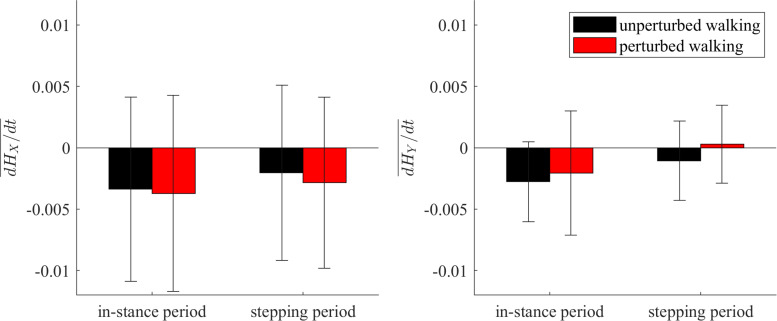
Dimensionless group-average and standard deviations of dH/dt summed over the in-stance and stepping periods. Black corresponds with the unperturbed experimental condition, red with the perturbed experimental condition.

Figure 9 shows the group average and standard deviations for the changes of whole-body angular momentum averaged over the in-stance and stepping periods. There were no statistically significant differences between both experimental conditions in neither of the tested periods.

### Ankle Kinematics

**FIGURE 10:**
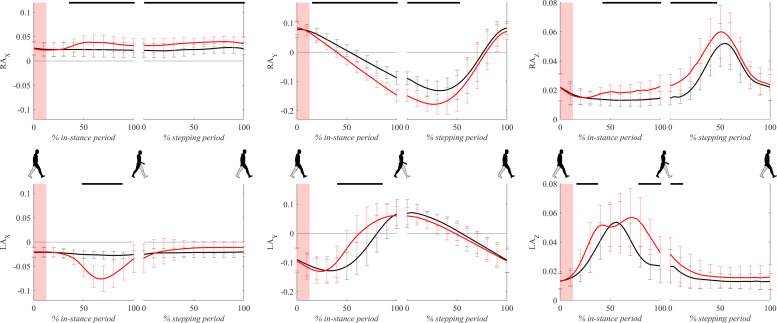
Dimensionless group-average ankle trajectories (medial malleolus) of the right leg (RAx, RAy, and RAz) and the left leg (LAx, LAy, and LAz) after outward perturbations (*N* = 11). Standard deviations are shown at each 10% of a gait cycle. Black corresponds with the unperturbed experimental condition, red with perturbed experimental conditions. One gait cycle divided into the in-stance and stepping periods, normalized to its duration separately for each experimental condition, is shown following a perturbing push. To visualize normalization of both periods short discontinuity is introduced on abscise. The perturbation period is marked with the red bar. Periods where statistically significant changes were determined between both experimental conditions are indicated with piece-wise continuous black lines on top of each graph.

Figure 10 shows all three components of ankle trajectories for the right and the left leg for both experimental conditions for two gait cycles. There was a significant difference in the frontal plane component (*x* axis) of the right ankle position from 40 to 100% of the in-stance period. Significant differences in the sagittal plane component (*y* axis) and vertical component (*z* axis) of the right ankle position can be seen throughout the large parts of in-stance and stepping periods.

A significant displacement of the left ankle position can be seen in the frontal plane component (*x* axis) and vertical component (*z* axis) in the second part of the in-stance period, which was related to a marked abduction of the left leg (Supplementary Video S1). A significant difference can also be observed in the frontal plane component (*x* axis) in the second part of the stepping period. This difference showed that following the perturbation the next step made by the left leg was on average placed slightly in the direction of the perturbation indicating limited use of the “stepping strategy.” The sagittal plane component (*y* axis) of the left ankle position showed in terms of peak-to-peak displacement substantial similarity between both experimental conditions suggesting substantially similar step lengths.

## Discussion

The results of this study show that the in-stance dynamic balancing responses following a moderate outward perturbation applied during the double stance during very slow walking simultaneously fulfill the following three objectives:

1. Counteract the action of perturbation by decelerating COMx very early in the stance phase through application of “ankle and inertial strategies” manifested in a pronounced GRFx impulse.
2. Adjust the movement of COMy in the sagittal plane first by application of the “braking strategy” in the first part of the stance followed by a propulsive action of GRFy in the second part of the stance phase such that at the end of the gait cycle COMx and COMy are synchronized to the values seen in unperturbed walking.
3. Maintain control of whole-body angular momentum both in frontal and sagittal planes by adequately modulating COP and GRF throughout the stance phase immediately after the perturbation commenced.

### Interplay of “In-Stance” Balancing Strategies

We investigated balancing strategies that primarily change the COP and GRF under the leg in stance following an outward perturbation as well as the governing principles of their interaction which has been so far to a large extent unknown (Bruijn and van Dieen, 2018). Several studies have demonstrated tight control of angular momentum during unperturbed walking (Herr and Popovic, 2008) or walking on a narrow beam (Chiovetto et al., 2018). A study from Sheehan et al. (2015) has demonstrated that tight control of angular momentum was also important during walking on a surface that was continuously perturbed laterally. The results of this study show that control of angular momentum is also a very important objective for very slow walking perturbed in the outward direction. However, this control is not very tight. In order to adequately control angular momentum in a gait cycle following the application of an outward perturbation well-tuned interaction between the “inertial strategy,” “medio-lateral ankle strategy,” and “braking strategy” was required.

Our first hypothesis stated that an impulse-like increase of GRFx that rapidly acts against an outward perturbation (Matjačić et al., 2019) would be accompanied by concomitant changes in GRFz, GRFy, COPx, and COPy that according to the model presented in Figure 1 act to maintain tight regulation of the overall angular momentum around the COM in the sagittal and frontal planes. The results show that the simple model suggested in Figure 1 explains regulation of angular momentum following an outward perturbation only partially. The “inertial strategy,” consisting from a short burst of GRFx impulse that opposed to the perturbation also substantially increased the angular momentum change in the frontal plane (van den Bogaart et al., 2020). Consistently with the prediction from Figure 1 this was initially accompanied with increase in COPx (“medio-lateral strategy”), COPy and GRFz, which, however, could not adequately balance the angular momentum change in the first part of the response (0–40% of the in-stance period). The angular momentum change in the frontal plane was balanced only in the second part of the response (40–100% of the in-stance period) when GRFx was substantially reduced.

Our second hypothesis stated that movement in the direction of progression would be temporarily slowed due to the anticipated increase in GRFy, which has been confirmed. Additionally, as a consequence of “braking strategy” also a notable angular momentum change has been introduced in the first part of the response in the sagittal plane, primarily due to the substantial anterior displacement of COPy. The prolonged duration of the stance phase following the perturbation enabled balancing of the angular momentum change in the sagittal plane in the second part of the response (Honeine et al., 2014).

The results of this study showed coupling of “medio-lateral” and “braking” balancing strategies, consistently with the proposition outlined in Figure 1B, which seem to be primarily in a function of controlling of angular momentum changes in both planes throughout the entire in-stance period. These angular momentum changes were caused by the application of “inertial” strategy, which was the primary response to the action of an outward perturbation applied at the heel strike in very slow walking as shown previously (Matjačić et al., 2019).

### The “Braking Strategy” and Related Subsequent Propulsion of COM in the Direction of Movement Progression

The experimental results of this study suggest that temporary deceleration of forward velocity following an outward perturbation during very slow walking has two distinct purposes. The first purpose is related to the increased deviation in COMx, caused by the action of perturbation, which requires more time to return to unperturbed values. Thus, slowing the movement in the plane of progression in the first part of the response is necessary to accommodate the increased deviation of COM in the frontal plane. Later, in the second part of the response COMy is accelerated forward ensuring the re-synchronization of pelvis movement in sagittal and frontal planes to the values seen in the unperturbed walking. The second purpose of slowing the movement in the sagittal plane in the first part of the response was to increase COPy which according to the Figure 1B facilitates further lateral displacement of COPx. However, this caused change in the angular momentum in the sagittal plane in the first part of the response, which was effectively balanced in the second part of the response due to the propelling action of increased GRFy and related increase in COPy-COMy (Honeine et al., 2014). Thus, it seems that the braking and consequent propelling action in the sagittal plane following outward perturbation have a self-regulatory effect on the maintenance of whole-body angular momentum in the sagittal plane during very slow walking.

### Modulation of GRFz

Contrary to the prediction of the simple mechanism presented in Figure 1 the experimental results of this study showed that even though GRFz was increased significantly immediately following the perturbation this increase was limited both in amplitude and duration. The limited increase in GRFz amplitude was expected as explained in the Introduction. Furthermore, very soon GRFz was reduced below the values observed during unperturbed walking thus controlling the vertical displacement of COMz.

### Duration of GRFx Impulse

A GRFx impulse of relatively short duration was used to predominantly counteract perturbation. One could ask why not prolong the duration of this force impulse, which could then even more efficiently counteract an outward perturbation. Our experimental results indicate that the answer could be related to the limited possibilities of increasing GRFz, probably to avoid reaching singularity in the knee, and to limited possibilities of displacing COPx laterally (Hof and Duysens, 2018). Thus, increasing the GRFx impulse even further would just add to imbalance in frontal plane angular momentum change in the first part of the response. It seems that GRFx impulse duration must be carefully chosen to accommodate the balance of whole-body angular momentum changes in the sagittal and frontal planes throughout the gait cycle following the perturbation.

### Relevance of the “Medio-Lateral Ankle Strategy” During Very Slow Walking

Hof (2007), Hof et al. (2010), and Hof and Duysens (2018) have suggested that the “medio-lateral ankle strategy” may not substantially counteract an outward perturbation while walking at normal speed due to the limited possibilities of lateral displacement but it is nevertheless important as it can act rapidly. The “medio-lateral ankle strategy” combined with concomitant antero-posterior displacement of COP following an outward perturbation during very slow walking as shown in this study does not only have the role of increasing GRFx as previously explained by Hof (2007) but crucially determines to what extent GRFx may be increased in the first part of the response and to what extent it must be decreased in the second part of the response. The larger the COPx displacement throughout the stance phase is, the easier the task of balancing whole-body angular momentum is in the frontal plane.

In the first part of the response a temporary increase in COPy facilitated larger COPx lateral displacement. In the second part of the response ankle eversion lifted the ankle upward 3 cm and thereby displaced COPx further laterally taking advantage of the increased width of the base of support due to the sole of the footwear. This can be seen in the right ankle trajectories in the *x* and *z* directions (Figure 10) where the ankle was shifted to the right and upward. Both mechanisms of shifting COPx laterally consecutively follow one another throughout the stance phase following perturbation. This may also have been related to the observed upward movement of COMz, which temporarily unloaded the stance leg thus facilitating the observed rotation of the foot in the frontal plane.

### Is Slow Walking More or Less Stable?

Orendurff et al. (2004) have found in their study that slow walking is accompanied by increased COM displacement in the frontal plane which necessitates more time to accomplish a step. This should also give more time to evaluate eventual perturbations thus consequently allowing more time to reposition the swinging leg through the “stepping strategy” which can then in the next stance phase act efficiently against perturbation. A similar proposition was made by Stimpson et al. (2018). The conclusion of both studies was that slower walking may be favorable among clinical populations as it is safer in terms of falling due to allowing more time to decide where to position the next step. This is probably the case for unperturbed walking and for moderate speeds of walking. The results of our study, however, show that dynamic balancing responses following a perturbing push in the outward direction during very slow walking predominantly consist of the “medio-lateral ankle strategy,” the “inertial strategy,” and the “braking strategy” executed within the stance phase immediately after the commencement of perturbation. Thus, waiting for corrective action associated with the “stepping strategy” may be inefficient in these conditions. In this sense it appears that unexpected perturbations may be very challenging to cope with during the very slow walking which is characteristic for clinical populations with limited sensory-motor control (Raja et al., 2012). This assumption remains to be investigated in further studies.

### Blending of Various Balancing Strategies

In this study, we have examined dynamic balancing responses at a single treadmill speed (0.4 m/s) and after a moderate perturbing push (10% of body weight) that commenced during the double support phase in the outward direction. This was done in order to isolate experimental conditions where the in-stance response would be dominant with a limited “stepping strategy” contribution coming from the changed location of the next step. In our previous studies we have seen that by increasing the intensity of a perturbing push during slow walking (0.4 m/s) some subjects responded by pivoting on the stance leg and also by substantially modulating the location of the next step (Matjačić et al., 2018, 2019). Similar findings were also presented in the study from Vlutters et al. (2018a). Pivoting on the stance leg is particularly interesting as it, beside the suggested mechanism from Figure 1B, effectively increases further displacement of COPx. This has the potential effect of further increasing the GRFx needed to counteract the perturbation, but also on the increased capacity of GRFz to counteract the change of angular momentum caused by increased GRFx. Thus, the oscillation in the GRFz after the commencement of perturbation as seen in this study may also have the function of temporarily unloading the stance leg which enables pivoting of the stance leg if that becomes necessary. When walking speed is increased to 0.6 m/s the results of our previous study (Matjačić et al., 2019) as well as the results from Vlutters et al. (2018b) have shown that a combination of balancing strategies (medio-lateral ankle, inertial, braking, and stepping) is used to counteract the effects of an outward perturbation.

### Estimation of Changes in Angular Momentum

Several methods are used to estimate the COM position during walking (Gard et al., 2004). The simplest is the sacral marker method which estimates COM position by tracking a single marker placed on the sacrum, assuming that the reciprocal movement of arms and legs to a large extent cancel out, as demonstrated by Herr and Popovic (2008), and thereby minimally influence the location of instantaneous whole-body COM. The segmental analysis method which is considered to be the gold standard uses estimates of the position of each segmental COM used to calculate whole-body COM through weighted sums, assuming that individual segmental masses and segmental COM can be determined accurately and further it treats body segments as rigid objects. However, as Yang and Pai (2014) pointed out, the segmental analysis method involves many assumptions and approximations, which make the accuracy of this method questionable. Several studies have compared both methods. Gard et al. (2004) compared only vertical excursions of COM during walking at different speeds and have shown that the sacral marker method and segmental analysis method produced equivalent results. Similarly, studies from Yang and Pai (2014), and Jeong et al. (2018) have shown that both methods produce similar results in the estimation of vertical and fore-aft excursions of COM while the estimation of medio-lateral COM produces similar results during the first part of the stance phase while latter in the gait cycle both methods can differ by up to 2 cm. Yang and Pai (2014) have additionally shown that the sacral marker method can reasonably estimate whole body COM during normal gait and slip. The studies from Jeong et al. (2018), and Yang and Pai (2014) have investigated the similarity of both methods at self-selected walking speed (around 1.2 m/s). Gard et al. (2004) have shown that as the walking speed is reduced the fit of both estimation methods is considerably increased. The lowest walking speed that they considered was 0.8 m/s. In our study the walking speed was much lower thus we may assume that the sacral marker method produced reliable COM estimates. Additionally, in our study we were not interested in absolute values for changes of whole-body angular momentum but rather in relative comparison between perturbed and unperturbed experimental conditions. Thus, given that no noticeable trunk and arm movement deviations between perturbed and unperturbed walking were observed (see the Supplementary Video S1), except for the increased lateral outward displacement of the swinging leg, which was accounted for in the COMx estimate as described in Methods, the potential errors made in the estimation of COM should be substantially similar for both experimental conditions (Major et al., 2018).

### Methodological Considerations

The balance assessment robot BART was controlled such that the interaction forces between the walking subject and pelvis link were as low as possible. We have assessed interaction forces in a previous study and found that the influence of these forces on COP and GRF in the sagittal and frontal planes as well as on the EMGs of major lower limb muscles during unperturbed walking had negligible effects in walking speeds ranging from 0.4 to 0.8 m/s (Olenšek et al., 2017). In another study we have demonstrated that the interaction between the balance assessment robot and the pelvis of a walking subject is purely passive, meaning that there is no exchange of energy between a walking subject and BART except for the period when a perturbing push is delivered (Matjačić et al., 2017). Thus, we may conclude that the method used to deliver outward perturbations to the pelvis of a walking subject had negligible effect on the presented results.

Our experimental protocol included not only outward perturbations of moderate strength and commencing at 0% of the gait cycle but also outward and inward perturbations commencing at various instants of gait cycle and at various perturbation strengths. In this way, we prevented possible anticipatory adjustments, for example leaning more toward the contralateral leg, in order to minimize the effect of the outward perturbation, which could have taken place if only one type of perturbation was delivered. The results of our previous study (Matjačić et al., 2019) have shown that such methodological approach prevented anticipatory adjustments, which increases the strength of our findings.

## Conclusion

In this study, we have revealed the mechanism of interaction between the three in-stance balancing strategies (“medio-lateral ankle strategy,” “inertial strategy,” and “braking strategy”), which act synergistically to efficiently counteract the effects of an outward directed push to the waist of humans walking very slowly on a treadmill. At the same time their action is such that changes of whole-body angular momentum are controlled and balanced in the frontal and sagittal planes within the in-stance period of gait cycle.

## Data Availability Statement

The datasets used and/or analyzed during the current study are available from the corresponding author on request for reasonable use.

## Ethics Statement

Ethical approval for this study was obtained from Republic of Slovenia National Medical Ethics Committee, decision number 80/03/15. All participants gave signed, written, informed consent. Participants gave consent to use and publish data in such way that anonymity is assured.

## Author Contributions

ZM conceived the draft structure and wrote the final version of the manuscript. MZ and AO critically revised the draft manuscript and contributed to all sections. ZM, MZ, and AO contributed substantially to the analysis of the data. MZ did most of the data processing and prepared the figures. All authors read and approved the final manuscript.

## Conflict of Interest

The authors declare that the research was conducted in the absence of any commercial or financial relationships that could be construed as a potential conflict of interest.

## Supplementary Material

VIDEO S1 In this video a balancing response to an outward-directed perturbing push is shown.

## Abbreviations

COM (COMx, COMy and COMz): vector of the whole-body center-of-mass position
COP (COPx, COPy and COPz): vector of the center-of-pressure position
dH/dt (dHx/dt, dHy/dt): vector of the whole-body angular momentum change
GRF (GRFx, GRFy and GRFz): vector of the ground-reaction-force
H (Hx, Hy): vector of the whole-body angular momentum (w. r. t. COM)
LA (LAx, LAy and LAz): vector of the left ankle marker (medial malleolus) position
RA (RAx, RAy and RAz): vector of the right ankle marker (medial malleolus) position.
